# Molecular characterization and induced changes of histone acetyltransferases in the tick *Haemaphysalis longicornis* in response to cold stress

**DOI:** 10.1186/s13071-024-06288-4

**Published:** 2024-05-12

**Authors:** Tingwei Pei, Miao Zhang, Ziwen Gao, Lu Li, Ziyan Bing, Jianglei Meng, Chuks Fidel Nwanade, Chaohui Yuan, Zhijun Yu, Jingze Liu

**Affiliations:** 1 Hebei Key Laboratory of Animal Physiology, Biochemistry and Molecular Biology, Hebei Collaborative Innovation Center for Eco-Environment, Hebei Research Center of the Basic Discipline of Cell Biology, Ministry of Education Key Laboratory of Molecular and Cellular Biology, College of Life Sciences, Hebei Normal University, Shijiazhuang, 050024 China; 2 The Professional and Technical Center of Hebei Administration for Market Regulation, Shijiazhuang, 050024 China

**Keywords:** *Haemaphysalis longicornis*, Epigenetics, Acetyltransferase, Cold response

## Abstract

**Background:**

Epigenetic modifications of histones play important roles in the response of eukaryotic organisms to environmental stress. However, many histone acetyltransferases (HATs), which are responsible for histone acetylation, and their roles in mediating the tick response to cold stress have yet to be identified. In the present study, HATs were molecularly characterized and their associations with the cold response of the tick *Haemaphysalis longicornis* explored.

**Methods:**

HATs were characterized by using polymerase chain reaction (PCR) based on published genome sequences, followed by multiple bioinformatic analyses. The differential expression of genes in *H. longicornis* under different cold treatment conditions was evaluated using reverse transcription quantitative PCR (RT-qPCR). RNA interference was used to explore the association of HATs with the cold response of *H. longicornis*.

**Results:**

Two HAT genes were identified in *H. longicornis* (Hl), a GCN5-related N-acetyltransferase (henceforth *HlGNAT*) and a type B histone acetyltransferase (henceforth* HlHAT-B*), which are respectively 960 base pairs (bp) and 1239 bp in length. Bioinformatics analysis revealed that HlGNAT and HlHAT-B are unstable hydrophilic proteins characterized by the presence of the acetyltransferase 16 domain and Hat1_N domain, respectively. RT-qPCR revealed that the expression of *HlGNAT* and *HlHAT-B* decreased after 3 days of cold treatment, but gradually increased with a longer period of cold treatment. The mortality rate following knockdown of* HlGNAT* or* HlHAT-B* by RNA interference, which was confirmed by RT-qPCR, significantly increased (*P* < 0.05) when *H. longicornis* was treated at the lowest lethal temperature (− 14 °C) for 2 h.

**Conclusions:**

The findings demonstrate that HATs may play a crucial role in the cold response of *H. longicornis*. Thus further research is warranted to explore the mechanisms underlying the epigenetic regulation of the cold response in ticks.

**Graphical Abstract:**

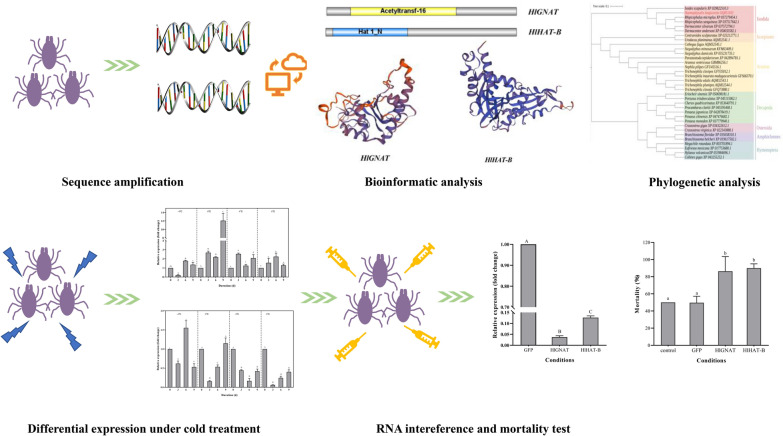

**Supplementary Information:**

The online version contains supplementary material available at 10.1186/s13071-024-06288-4.

## Background

Ticks are blood-sucking ectoparasites that can feed from a wide range of hosts and act as vectors for a large number of pathogens of concern for human and veterinary medicine [1–3]. Ticks have a complex life cycle involving different periods on-host and off-host. Cold stress is one of the major environmental factors that can affect the survival of ticks off-host. However, ticks have developed excellent tolerance to cold to overcome the negative effects of this stressor [4]. A series of biochemical changes, including changes in water, glycerol, fat, and protein contents, are related to cold tolerance in ticks [4, 5]. Recent studies have revealed the involvement of diverse genes, including those of the aquaporin, ferritin, superoxide dismutase, heat shock protein, and cold shock domain protein families, in the cold response of ticks [6, 7]. Epigenetic regulation by DNA methylation may also play an important role in the cold resistance of ticks [8, 9].

In addition to DNA methylation, numerous other forms of epigenetic regulation, including nucleosome localization, chromatin remodeling, histone modification, variable RNA splicing, noncoding RNA, RNA modification, and pseudogene regulation, have been identified [10]. Among them, histone modifications are widely recognized as dynamic regulators of transcription in environmental signal responses, and usually involve acetylation, methylation, phosphorylation, and ubiquitination of lysine or arginine residues at the free N-terminal tail region of histones H3 and H4 [11, 12]. Histone acetylation is catalyzed mainly by histone acetyltransferases (HATs) and histone deacetylases. HATs are typically responsible for catalyzing the acetylation of lysine in the N-terminal tail region associated with gene expression [13]. Generally, histone acetylation is mostly associated with transcriptional activation. Histone acetylation leads to changes in the positive electrical properties of lysine, which relaxes the chromatin structure and promotes the activation of gene transcription [14–16]. HATs are characterized by a conserved core that binds acetyl coenzyme A and a catalytic mechanism that transfers the acetyl portion of acetyl coenzyme A to its target [17–20]. The effector molecule recognizes the acetylated histone lysine and transmits the signal to downstream molecules [21].

Histone acetylation is known to be involved in the regulation of the stress response in organisms [22]. Vernalization of *Arabidopsis thaliana* can promote the binding of the protein complex VRN3 (normalization sensitivity 3) to VRN2 (normalization sensitivity 2), which may induce the binding of histone deacetylases and histone methyltransferases to the* FLC* gene, leading to the suppression of* FLC* gene expression; this phenomenon lasts until the flowering stage [23]. HAT- and histone deacetylase-mediated chromatin modifications play important regulatory roles in the survival of goldenrod gall fly *Eurosta solidaginis* and goldenrod gall moth *Epiblema scudderiana* under zero-temperature conditions [24]. The histone acetyltransferase Elp3 in the red flour beetle *Tribolium castaneum* plays an important role in its adaptation to environmental conditions under oxidative stress [25]. There is some knowledge on the functions of histone modifications in response to cold stress in plants [26], but the roles of histone modifications in mediating the response to cold stress in ticks have yet to be determined.

The Asian longhorned tick *Haemaphysalis longicornis* is widespread in China, and it is an invasive species in Australia, New Zealand, and the USA. Since *H. longicornis* was first discovered in the USA, in 2017, it has spread to 12 states in the country [27]. The range expansion of *H. longicornis* is particularly concerning for livestock farming as heavy infestations can kill animals due to blood loss, and the pathogens that this tick spreads are also a threat to livestock health [28, 29]. Therefore, understanding the mechanisms underlying the adaptation of *H. longicornis* to environmental factors related to cold stress is becoming increasingly important. In the present study, HATs were characterized and their expression under different cold treatment conditions evaluated via reverse-transcription quantitative polymerase chain reaction (RT-qPCR). RNA interference (RNAi) was used to explore the associations of the HATs with the cold response in *H. longicornis*. These results reported here should help us to further our knowledge on the epigenetic and molecular regulatory mechanisms involved in the cold response of ticks as a basis for the integrated control of ticks and tick-borne diseases.

## Methods

### Tick feeding and sample preparation

Bisexual ticks of *H. longicornis* were collected by flag-dragging  in the Xiaowutai National Nature Reserve Area (39°50–40°07′N, 114°47–115°30′E), Hebei Province, China. Nonparasitic ticks were placed in 5-mL Eppendorf tubes in a laboratory incubator under controlled conditions (temperature 26 ± 1 °C, relative humidity 75 ± 5%, 16:8-h light:dark), and they were placed on the ears of New Zealand white rabbits for feeding. Second-generation unfed adult female ticks that had molted for approximately 2 weeks were randomly selected for subsequent assays. All the animal feeding experiments were approved by the Animal Ethics Committee of Hebei Normal University [Institutional Animal Care and Use Committee (IACUC) protocol number 207908].

### RNA extraction and complementary DNA synthesis

Twenty unfed female *H. longicornis* were placed in a 1.5-mL Eppendorf tube and sterilized with double-distilled (dd) H_2_O and 75% ethanol. The ticks were then ground in liquid nitrogen, and total RNA was extracted using TRIzol (Invitrogen, Carlsbad, CA) following the manufacturer’s protocol. RNA quantification was performed using a NanoDrop ND-2000 Spectrophotometer (Thermo Fisher Scientific, Waltham, MA), with the A260/A280 ratio generally above 2.0, and RNA quality was assessed by 1% agarose gel electrophoresis.

Complementary DNA (cDNA) was synthesized using TransScript One-Step gDNA Removal and cDNA Synthesis SuperMix (TransGen, Beijing) according to the manufacturer’s instructions. Briefly, a total of 20 µL of mixture was used, which included 1–7 µL of total RNA, 1 µL of Oligo (dT) Anchored 18 Primer (0.5 μg/µL) , 10 µL of 2× TransScript Reaction Mix, 1 µL of EasyScript RI Enzyme Mix, 1 µL of gDNA Remover, and 0–6 µL of RNase-free water. The PCR conditions were as follows: 42 °C for 30 min, 85 °C for 5 s. The primers (Table 1) were designed using Primer Premier version 5.0 for Windows (Premier Biosoft International, Palo Alto, CA) based on the genomic sequences (Bio-Project PRJNA668644) [6]. The PCR cycling parameters were as follows: initial denaturation at 94 °C for 2 min; 40 cycles at 94 °C for 30 s, 30 s at a melting temperature of 60 °C, and 30 s at 72 °C; and a final extension at 72 °C for 10 min on an Applied Biosystems Veriti 96-well Thermal Cycler (Life Technologies, Marsiling, Singapore). The amplified fragments were verified and separated on a 1% agarose gel. Bands with the expected sizes were excised and purified with an EG101-01 EasyPure Quick Gel Extraction Kit (TransGen) according to the manufacturer’s protocol. The purified products were sequenced and used for subsequent analysis.

**Table 1:** Primers designed for genes encoding histone acetyltransferases of* Haemaphysalis longicornis*

Methods	Gene	Primer (5′-3′)
Polymerase chain reaction (PCR)	*HlGNAT*	Forward: ATGGAATTTCCATTCAGTGTAGC
		Reverse: TCAGTCCAAGCTAGCCCG
	*HlHAT-B*	Forward: ATGAAAATGGCGGGTGTT
		Reverse: TCAGCTACTCGGAGCAGC
Reverse transcription quantitative PCR	*HlGNAT*	Forward: GAGCAGTTCAACGCCACCA
		Reverse: GGGAAACACGGCAAAATG
	*HlHAT-B*	Forward: CTGCGAGACTTCGTGGACT
		Reverse: GCTGGCATTTGCTGTGTTAG
	Beta-actin (*ACTB*)	Forward: CGTTCCTGGGTATGGAATCG
		Reverse: TCCACGTCGCACTTCATGAT
RNA interference	*HlGNAT*	Forward: TCAGCCCTCGGAGAAGAT
		Reverse: GCGGTGGTAAGCCAAAGA
	*HlHAT-B*	Forward: ATGGCGGGTGTTTCGTTG
		Reverse: GGGCTCTTTAGGCAGGATTG

### Bioinformatic analysis

Sequence alignment and identity analyses were performed with DNAMAN (Lynnon Biosoft, San Ramon, CA) and Basic Local Alignment Search Tool (BLAST) for nucleotides [National Center for Biotechnology Information (NCBI) http://www.ncbi.nlm.nih.gov/BLAST]. The phylogenetic relationships of HATs among different species were constructed using MEGA11 software (http://www.megasoftware.net/), and the evolutionary tree was constructed with the iTOL web tool (https://itol.embl.de/). The conserved domains were constructed using the Conserved Domain Database on the NCBI website and with Illustrator of Biological Sequence v1.0. The physicochemical properties of the corresponding proteins were analyzed and predicted through Expert Protein Analysis System (Expasy) (http://www.expasy.org), the DiAminoacid Neural Network Application (DIANNA) website (http://bioinformatics.bc.edu/clotelab/DiANNA/), and BioEdit software (http://www.mbio. ncsu.edu/BioEdit/BioEdit.html). The secondary and tertiary structures of HATs were predicted using the self-optimized prediction method (SOPMA) (https://npsa-prabi.ibcp.fr/cgi-bin/npsa_automat.pl?page=/NPSA/npsa_sopma.html) and the Swiss Model website (https://swissmodel.expasy.org/), respectively.

### Relative expression of HAT genes in *H. longicornis* under cold treatment

Ticks of *H. longicornis* were treated at different low temperatures (− 4 °C, 0 °C, 4 °C, 8 °C) under the same humidity conditions as the control group for 3, 6, and 9 days, with ticks maintained at 26 °C serving as the control. Each treatment group comprised 20 unfed females, and each treatment was replicated three times at each treatment temperature. After treatment, the groups of ticks were immediately ground in liquid nitrogen, and RNA was extracted as described above. The relative expression of HAT genes under different cold treatments was determined using RT–qPCR. Briefly, 20 μL of standard PCR mixture was amplified with 1 μL of the cDNA synthesized as described above, 0.4 µL of gene-specific primers (forward and reverse), 0.4 μL of Passive Reference Dye II, 7.8 μL of RNase free H_2_O, and 10 µL of 2× TransStart Top Green qPCR SuperMix (TransGen). The conditions were set as follows: initial 30-s denaturation at 94 °C; 40 cycles of 5 s at 94 °C, 30 s at 60 °C, and 1 min at 95 °C; and a final extension at 55 °C for 30 s, and 95 °C for 30 s. The beta-actin (*ACTB*) gene was used as a reference because it has been successfully used as a reference gene in other tick stress studies [30]. The 2^−ΔΔCt^ relative quantification method, in which beta-actin was used for normalization, was used to estimate target gene expression; the figures were generated with GraphPad Prism 8.0 software (GraphPad, San Diego, CA).

### RNA interference

For double-stranded RNA (dsRNA) synthesis, the T7 promoter sequence (5' TAATACGACTCACTATAGG 3') was added to the 5′-end of each primer. Briefly, two reaction solutions were prepared. The first solution (40 µL) contained 2 µL of cDNA, 16 µL of RNase-free water, 20 µL of 2× TransStart MasterMix (dye), 1 µL of forward primer and 1 µL of T7-Reverse primer. The second solution (20 µL) contained 1–8 µL of cDNA, 7–0 µL of RNase-free water, 10 µL of 2× Express Buffer, and 2 µL of T7 Express Mix. After incubation at 37 °C for 30 min, the two solutions were mixed and incubated at 70 °C for 20 min. Then, 2 µL of RQ1 RNase-free DNase and 2 µL of RNase A solution was added and the mixture incubated at 37 °C for 30 min. Finally, 4.4 µL of sodium acetate and 110 µL of 95% ethanol were mixed, incubated until ice cold for 5 min, and centrifuged at 16,000 × *g* for 5 min; the supernatant was removed, and diethyl pyrocarbonate-treated water was added, with the final concentration of dsRNA adjusted to 8000 ng/µL. Groups of ticks (20 unfed females) were then sequentially sterilized with ddH_2_O, H_2_O_2_, and ddH_2_O. One microliter of dsRNA was injected into the third and fourth coxa using Microliter syringes (Hamilton, NE), and the control group was injected with 8000 ng of green fluorescent protein (GFP) dsRNA. The RNA sequence of the GFP-encoding gene served as a control dsRNA that could be used to control for any changes in gene expression that may have been caused by the induction of other genes. Each treatment was replicated three times. The ticks were then placed in a laboratory incubator (temperature 26 ± 1 °C, relative humidity 75 ± 5%, 16:8-h light:dark) for 24 h to recover. The relative expression of the target genes after knockdown was confirmed by RT-qPCR, as described above.

After confirmation of the knockdown of the target genes, groups of ticks (each comprising 20 unfed females) injected with dsRNA from *gfp*, *HlGNAT* or *HlHAT-B*, were exposed to a lower lethal temperature of − 14 °C for 2 h [4]; uninjected ticks served as the control group. Each treatment was replicated three times. Then the ticks were removed from the thermostatic bath and placed in a laboratory incubator (temperature 26 ± 1 °C, relative humidity 75 ± 5%, 16:8-h light:dark) for 24 h of recovery. Ticks without body movement or with no leg coordination under CO_2_ stimulation were considered dead for calculation of the mortality rate.

### Statistical analysis

Multiple comparisons between the control and treatment groups were performed using an unpaired* t*-test with GraphPad Prism software, version 8.0. A *P*-value < 0.05 was considered to indicate statistical significance.

## Results

### Molecular characterization of HAT genes of *H. longicornis*

**Fig. 1A–C:**
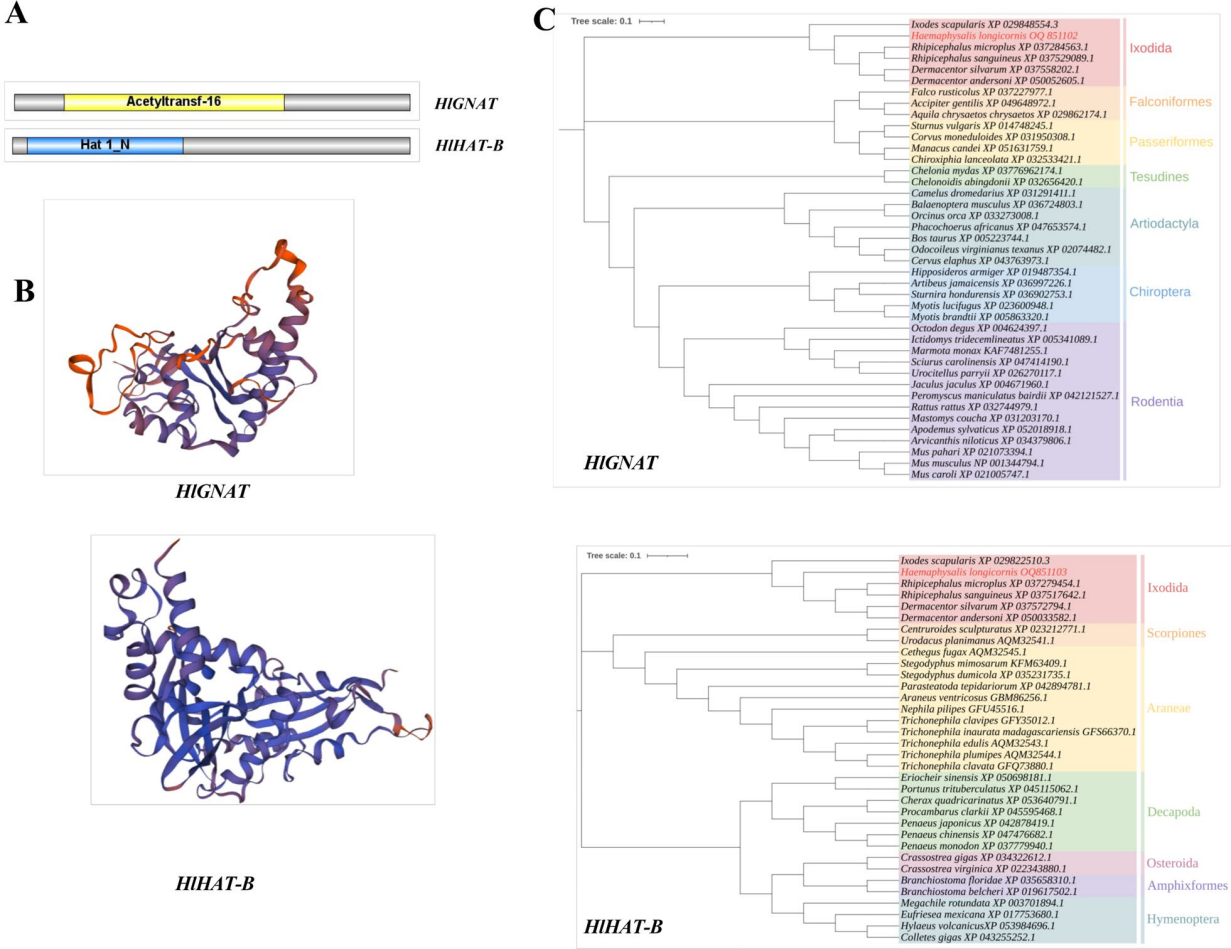
Bioinformatic analysis of the HATs characterized from *Haemaphysalis longicornis*. **A** Schematic of the conserved domain; **B** the predicted tertiary structures; **C** phylogenetic tree of the amino acid sequences

*HlGNAT* and *HlHAT-B*, which have lengths of 960 bp and 1239 bp, respectively, were cloned from the tick *H. longicornis* (Additional file 1: Fig. 1). The sequences were deposited in the NCBI database under accession numbers OQ851102 and OQ851103, respectively. Analysis via BLASTx (NCBI) showed that *HlGNAT* displayed 74.38% similarity with the alpha-tubulin N-acetyltransferase 1-like isoform × 2 from *Dermacentor silvarum*, whereas *HlHAT-B* displayed 88.59% similarity with the histone acetyltransferase type B catalytic subunit-like from *Rhipicephalus sanguineus* (Fig. 1). Phylogenetic analysis revealed that *HlGNAT* and *HlHAT-B* clustered with HAT genes from *D. silvarum*. *HlGNAT* and *HlHAT-B* have molecular weights of 35.74 kDa and 47.74 kDa, respectively. According to the Conserved Domains (NCBI) search, HlGNAT contains the acetyltransferase_16 domain and HlHAT-B contains the Hat1_N domain (Fig. 1).

The predicted physicochemical properties of HlGNAT and HlHAT-B indicated that the molecular formulas of *HlGNAT* and *HlHAT-B* are C1581H2485N469O462S9 and C2142H3325N569O634S17, respectively, and that these proteins are unstable and hydrophilic (Table 2). No signal peptide or transmembrane domains were detected in HlGNAT or HlHAT-B. PSORT II prediction revealed that HlGNAT was localized in the nucleus with a high probability of 73.9%, and in the cytoplasm with a probability of 39.1%, and that HlHAT-B was localized in the nucleus with a probability of 43.5% (Table 3). Both HlGNAT and HlHAT-B have a mixed secondary structure, with alpha helices and random coils accounting for a large proportion of these proteins, whereas the proportion of beta turns is relatively low (Table 4). The Global Model Quality Estimate values of HlGNAT and HlHAT-B were 0.45 and 0.66, respectively. Alpha A crystallin gave the best templates, with a high level of confidence for the three-level structure prediction models of HlGNAT and HlHAT-B (Fig. 1).

**Table 2:** Predicted properties of the histone acetyltransferases HlGNAT and HlHAT-B of* Haemaphysalis longicornis*

Physicochemical property	Proteins	
	HlGNAT	HlHAT-B
Total number of atoms	5006	6687
Molecular weight (kDa)	35.74	47.74
Theoretical isoelectric point	9.53	6.14
Hydrophobicity index	− 0.491	− 0.456
Instability index	67.42	43.33
Aliphatic index	73.67	80.46

**Table 3:** Predicted subcellular localizations of the histone acetyltransferases HlGNAT and HlHAT-B in *Haemaphysalis longicornis*

Subcellular localization	HlGNAT (%)	HlHAT-B (%)
Nucleus	73.9	43.5
Mitochondrion		13.0
Cytoplasm	17.4	39.1
Secretory vesicles		4.3
Peroxisome	8.7

**Table 4:** The secondary structure of the histone acetyltransferases HlGNAT and HlHAT-B in *Haemaphysalis longicornis*

HATs	Alpha helix (hh) (%)	Extended strand (ee) (%)	Beta turn (tt) (%)	Random coil (cc) (%)
HlGNAT	24.76	15.36	4.08	55.80
HlHAT-B	45.87	16.26	2.91	34.95

### Relative expression of *HlGNAT* and *HlHAT-B *in *H. longicornis* under cold treatment

RT–qPCR revealed that both *HlGNAT* and *HlHAT-B* were expressed throughout the treatment period, and that after cold treatment, the relative expression of these genes exhibited different trends at different treatment durations.

**Fig. 2:**
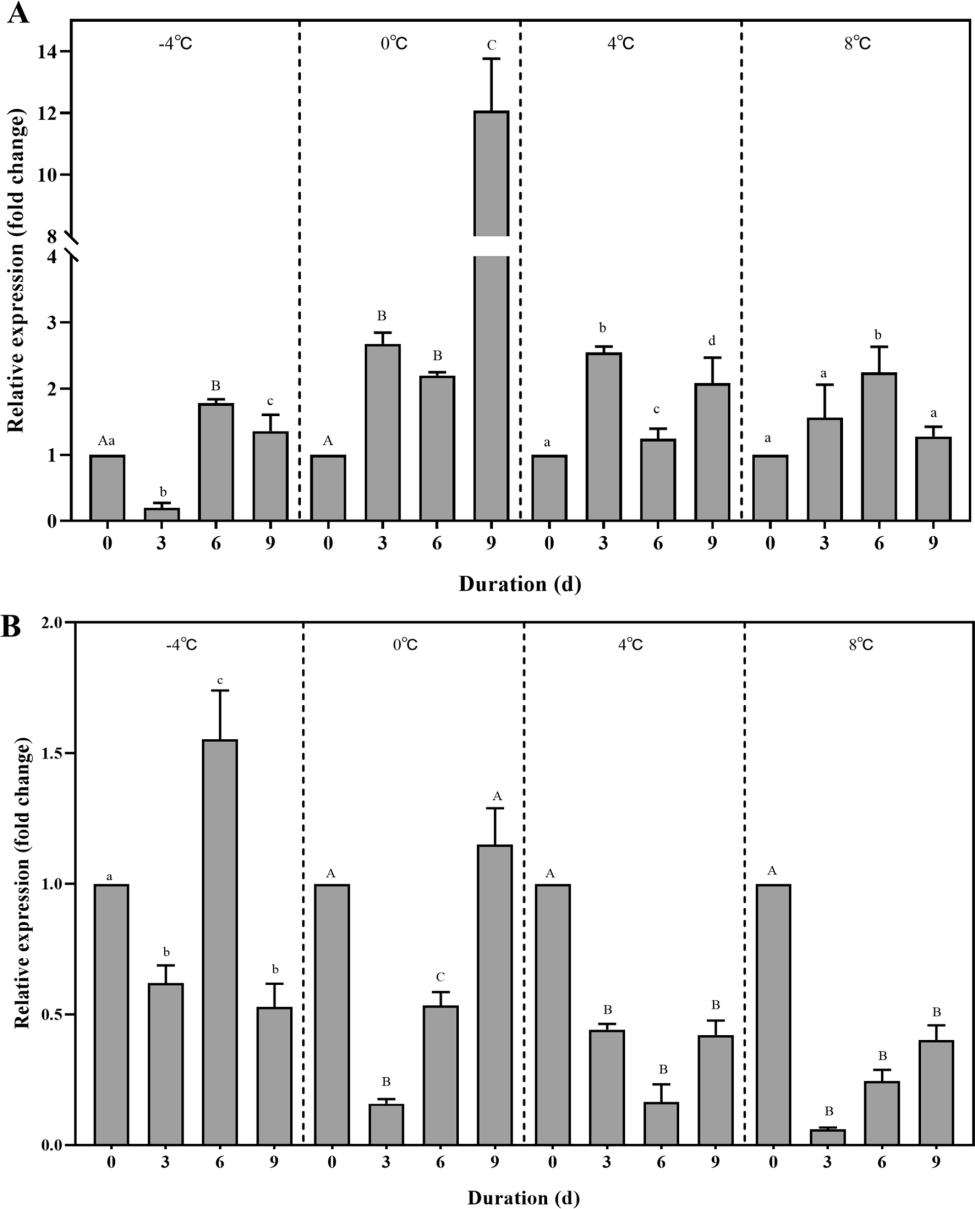
Relative expression of *HlGNAT* (**A**) and *HlHAT-B* (**B**) in *Haemaphysalis longicornis* exposed to cold treatments. **A**, **B** Different uppercase letters indicate significant difference at *P* < 0.01; different lowercase letters indicate significant difference at *P* < 0.05.* d* Day

At 0 °C and 4 °C, *HlGNAT* expression showed an upward trend within 3 days, followed by a downward trend; *HlGNAT* expression reached its lowest value on the 6th day (*P* < 0.01) and then showed an upward trend. At – 4 °C, there was a downward trend within 3 days, followed by an upward trend, with a peak on the 6th day (*P* < 0.01), and then a downward trend. After treatment at 8 °C, no significant changes were observed for 3 days, after which the expression tended to increase, peaking on the 6th day (*P* < 0.05) (Fig. 2A).

At − 4 °C, 0 °C, and 8 °C, *HlHAT-B* expression showed a downward trend for 3 days, reached its lowest value on the 3rd day (*P* < 0.05), and then showed an upward trend. At 0 °C and 8 °C, an upward trend was observed for the 6th to 9th days. After treatment at 4 °C, a downward trend was observed for 6 days, at which point the lowest value was reached (*P* < 0.01), after which the trend was upward (Fig. 2B).

### RNA interference

**Fig. 3A, B:**
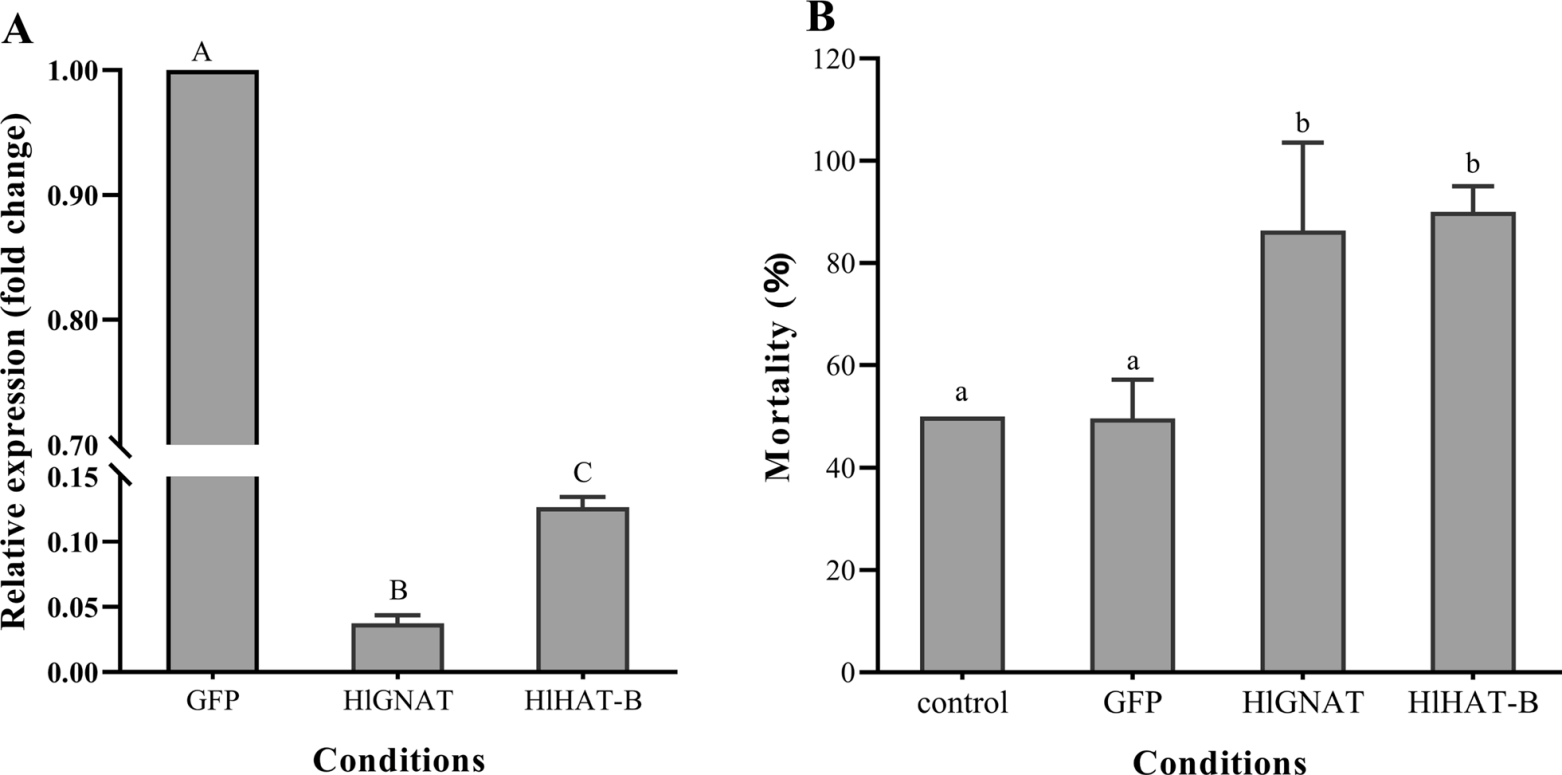
The effects of RNA interference (RNAi) on the cold tolerance of *Haemaphysalis longicornis*. **A** Relative expression of HAT genes after RNAi. **B** Mortality of *H. longicornis* under lower lethal temperature treatment after RNAi. Each treatment was replicated three times; there were 20 unfed females in each group. Different uppercase letters indicate significant difference at *P* < 0.01; different lowercase letters indicate significant difference at *P* < 0.05.* GFP* Green fluorescent protein

Following RNAi, the expression of both *HlGNAT* and *HlHAT-B* decreased significantly (*P* < 0.01). The average silencing efficiency for *HlGNAT* and *HlHAT-B* in *H. longicornis* was 96% and 88%, respectively, indicating that injection of the dsRNA of the HAT genes significantly inhibited the expression of the target genes (Fig. 3A).

Following RNAi, the ticks were treated at a lower lethal temperature of -14 °C for 2 h. The mortality rate was significantly higher (*P* < 0.05) in the groups treated with dsRNA from *HlGNAT *(86.33%) and * HlHAT-B* (90%) compared to the control (50%) and the group treated with dsRNA from GFP (49.67%), indicating that the expression of both *HlGNAT* and *HlHAT-B* was closely associated with the cold tolerance of *H. longicornis* (Fig. 3B).

## Discussion

Histone modifications and histone-modifying enzymes play pivotal roles in the regulation of transcription, which is highly dynamic in response to stress [31]. Several HAT genes have been cloned from species of ticks, including *D. silvarum*, *Ixodes scapularis,* and *Amblyomma maculatum* [32–34]. However, these genes belong to different gene families, and the functions in response to various environmental stresses of the histone-modifying enzymes that they encode are unclear. In the present study, two HAT genes, *HlGNAT* and *HlHAT-B*, were identified and characterized from *H. longicornis*, and their associations with the low-temperature response of this tick explored.

Predicting the physicochemical properties of proteins helps to extend our knowledge about their structure and function [35]. In the present study, we found that HlGNAT and HlHAT-B are unstable hydrophilic proteins with relatively high aliphatic indices, ranging from 70 to 80, which indicate high thermal stability. Both of these proteins are nonsecretory and do not contain transmembrane regions, according to the signal peptide prediction. The subcellular localization of HlGNAT and HlHAT-B showed that they were distributed between different parts of the cells, but were predominantly located in the nucleus, which confirms that they play a role in the acetylation of histones of chromosomes in this organelle [36].

On the basis of the structural homology and function of their catalytic domains, HATs can be divided into four categories: the GNAT family; the MYST family (Moz, Ybf2/Sas3, Sas2, Tip60); p300/CBP (CREB binding protein); and other, undifferentiated HATs [37, 38]. GNAT is further subdivided into histone acetyltransferase 1 (HAT1), KAT2A (GCN5), and KAT2B (PCAF) [39]. *HlGNAT* and *HlHAT-B*, found in the tick *H. longicornis*, belong to the GNAT family and the type B histone acetyltransferases (HAT-B), respectively. HAT-B is the only known member of the type B histone acetyltransferases that exhibits specificity toward free H4 [40]. Three HATs, *DsCREBBP*, *DsKAT6B* and *DsKAT5*, which belong to the p300/CBP (KAT protein family) have been characterized in *D. silvarum* [34]. Different expression patterns have been found for distinct categories of HAT genes in response to cold stress in ticks. In the present study, the expression of *HlGNAT* and *HlHAT-B* initially decreased after 3 days of cold treatment, but then gradually increased with a prolonged period of exposure of *H. longicornis* to cold. In contrast, the expression of *DsCREBBP*, *DsKAT6B*, and *DsKAT5* in *D. silvarum* initially increased in response to cold treatment before gradually decreasing [34].

In the present study, the expression of *HlGNAT* and *HlHAT-B* in *H. longicornis* exhibited different trends after cold treatment, indicating that HATs may play different roles in the cold response of ticks. Similar to the decreasing trend in the expression of *HlHAT-B* in *H. longicornis* under cold treatment found in the present treatment, total HAT activity was reported to decrease under cold treatment at 5 °C and − 15 °C in *E. scudderiana* [24]. Histone acetylation leads to a looser chromatin structure, which facilitates the binding of transcription factors and causes transcriptional activation. Under cold treatment in the present study, the overall expression level of *HlGNAT* tended to increase. Similar results were also observed for *E. solidaginis* treated at 5 °C for 3 weeks, in which the total HAT activity was 1.40 times that of the control group; after treatment at − 15 °C for 1 week, the total HAT activity was 1.47 times that of the control group [24]. This phenomenon might be explained by the maintenance of the transcriptional activity of HATs in a “standby” state during the freezing process, which allows metabolic activity to change rapidly in the event of rapid thawing. The increase in HAT activity may be related to the upregulation of enzyme-encoding genes/proteins by cryoprotectants (glycerol and sorbitol). In addition, HATs are known to be involved in the regulation of DNA repair and apoptosis [41, 42], and the potential effect of increased HAT activity may be selective, allowing the expression of certain genes, such as those involved in cell repair processes [24].

Under cold treatment, the expression of *HlGNAT* showed an obvious increase, whereas the changes in *HlHAT-B* expression were relatively weak. After the injection of dsRNA from *HlGNAT* and *HlHAT-B*, the mortality rate of ticks at different temperatures significantly increased, and their cold tolerance decreased. It is speculated that *HlGNAT* and *HlHAT-B* may play a role in the cold regulation of *H. longicornis*. However, there were several limitations in the present study that need to be addressed in future validation studies. Though beta-actin is widely used as an internal reference gene for RT-qPCR quantitation of gene expression, concerns have been raised regarding its stability in insects that are under stress [43]. Therefore, the stability of the expression of beta-actin under cold treatment of ticks should be determined, or alternative reference genes should also be used to confirm the changes in *HlGNAT* and *HlHAT-B*. Furthermore, factors beyond the direct effects of gene knockdown may have had an influence on the observed increase in tick mortality following RNAi, such as off-target effects or unintended physiological consequences [44]. One caveat with respect to RNAi is that knockdown of a HAT may lead to a decrease in survival even without cold exposure, though no obvious difference was found between the control group and the GFP-dsRNA group (*P* > 0.05). Determining the levels of the proteins *HlGNAT* and *HlHAT-B* may help to partly limit the discrepancies seen here between the effects of RNAi on tick mortality and the RT-qPCR results.

In summary, although this study provides molecular insights into the HATs of *H. longicornis*, further research is needed to overcome the limitations discussed above to further elucidate the epigenetic mechanisms involved in the cold response of ticks.

## Conclusions

Two genes, *HlGNAT* and *HlHAT-B*, encoding HATs were characterized from the tick *H. longicornis*. HlGNAT and HlHAT-B are unstable hydrophilic proteins characterized by the presence of the acetyltransferase 16 domain and the Hat1_N domain, respectively. The relative expression of *HlGNAT* and *HlHAT-B* varied with the duration of cold treatment. Knockdown of *HlGNAT* or *HlHAT-B* by RNAi significantly increased the mortality of *H. longicornis* after exposure to a lower lethal temperature (− 14 °C) for 2 h, which demonstrated that HATs may play a crucial role in the cold response of this species of tick.

### Supplementary Information

**Additional file 1: Figure S1.** The nucleotide and encoded amino acid sequence of HATs of* Haemaphysalis longicornis* (**A** *HlGNAT*, **B** *HlHAT-B*).

## Data Availability

The sequences have been deposited in the NCBI database under accession numbers OQ851102 and OQ851103.
