# Supplementary figures and images for: Molecular characterization and induced changes of histone acetyltransferases in the tick Haemaphysalis longicornis in response to cold stress
Source: Parasit Vectors. 2024 May 12;17:218. doi: 10.1186/s13071-024-06288-4 (PMC11089763; doi:10.1186/s13071-024-06288-4)

## Slide 1
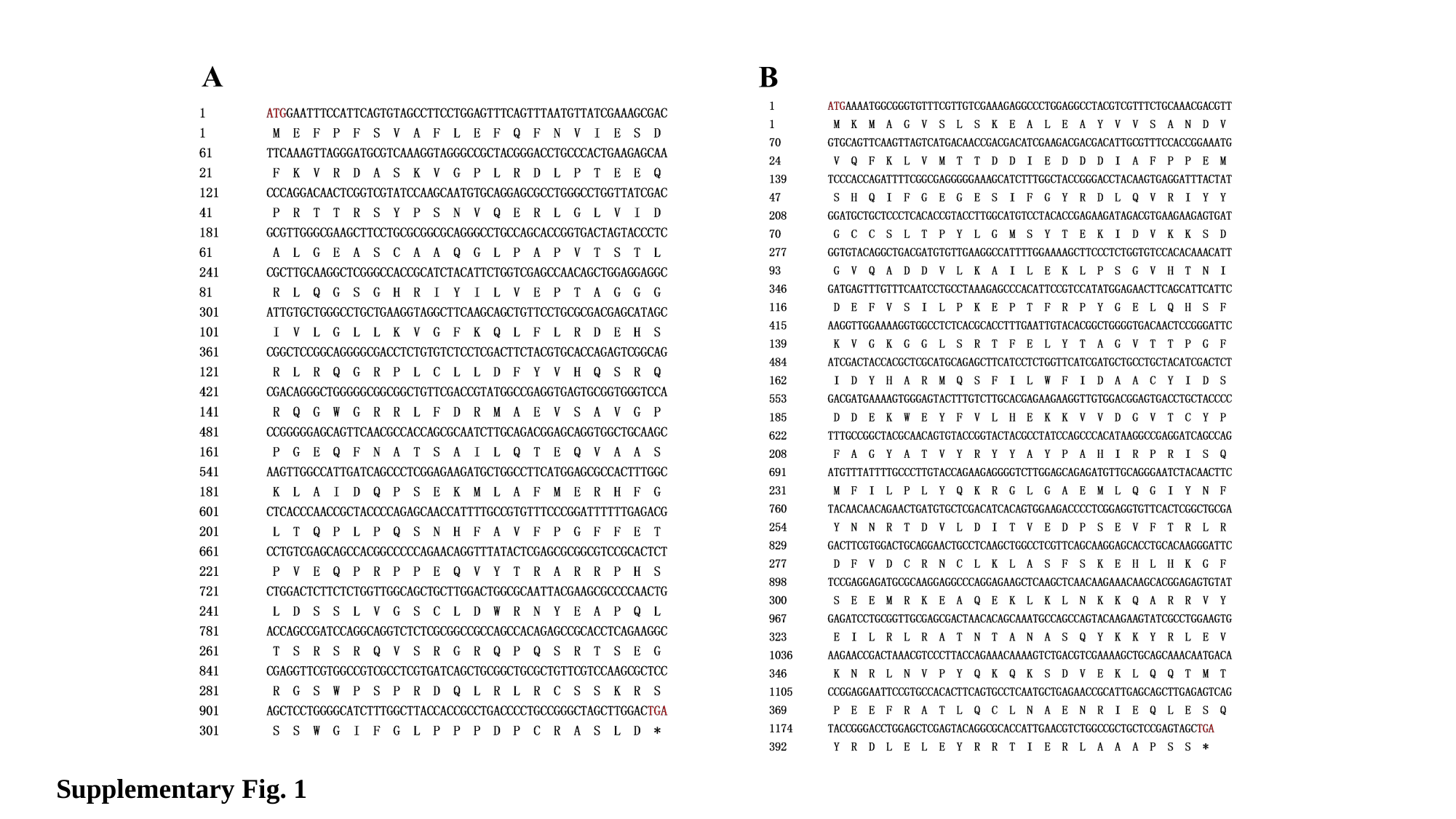

Supplementary Fig. 1

Supplement: Supplementary file 1 — Additional file 1: Figure S1. The nucleotide and encoded amino acid sequence of HATs of Haemaphysalis longicornis (A HlGNAT, B HlHAT-B). [file 13071_2024_6288_MOESM1_ESM.pptx]
